# Potential Mediating Role of Iron Biomarkers in the Association of Sex With Glucose, Insulin, and Type 2 Diabetes

**DOI:** 10.1210/jendso/bvae098

**Published:** 2024-05-16

**Authors:** Farnaz Khatami, Theis Lange, Dion Groothof, Noushin Sadat Ahanchi, Hugo G Quezada-Pinedo, Hamidreza Raeisi-Dehkordi, Martin H De Borst, Pedro-Marques Vidal, Sailesh Mohan, Dorairaj Prabhakaran, Arjola Bano, Stephan J L Bakker, Taulant Muka, Michele F Eisenga

**Affiliations:** Institute of Social and Preventive Medicine (ISPM), University of Bern, 3012 Bern, Switzerland; Graduate School for Health Sciences, University of Bern, 3012 Bern, Switzerland; Community Medicine Department, Tehran University of Medical Sciences, 1417613151 Tehran, Iran; Department of Public Health, Section of Biostatistics, University of Copenhagen, DK-1353 Copenhagen, Denmark; Division of Nephrology, Department of Internal Medicine, University Medical Center Groningen, University of Groningen, 9713 GZ Groningen, The Netherlands; Institute of Social and Preventive Medicine (ISPM), University of Bern, 3012 Bern, Switzerland; Graduate School for Health Sciences, University of Bern, 3012 Bern, Switzerland; Department of Medicine, Internal Medicine, Lausanne University Hospital and University of Lausanne, 1011 Lausanne, Switzerland; The Generation R Study Group, University Medical Center Rotterdam, 3000 CA Rotterdam, The Netherlands; Institute of Social and Preventive Medicine (ISPM), University of Bern, 3012 Bern, Switzerland; Julius Center for Health Sciences and Primary Care, University Medical Center Utrecht, Utrecht University, 3584 CX Utrecht, The Netherlands; Division of Nephrology, Department of Internal Medicine, University Medical Center Groningen, University of Groningen, 9713 GZ Groningen, The Netherlands; Department of Medicine, Internal Medicine, Lausanne University Hospital and University of Lausanne, 1011 Lausanne, Switzerland; Centre for Chronic Conditions and Injuries (CCCI), Public Health Foundation of India, 110070 Delhi, India; Centre for Chronic Disease Control (CCDC), 110016 Delhi, India; Centre for Chronic Conditions and Injuries (CCCI), Public Health Foundation of India, 110070 Delhi, India; Centre for Chronic Disease Control (CCDC), 110016 Delhi, India; Institute of Social and Preventive Medicine (ISPM), University of Bern, 3012 Bern, Switzerland; Department of Cardiology, Bern University Hospital, University of Bern, 3010 Bern, Switzerland; Division of Nephrology, Department of Internal Medicine, University Medical Center Groningen, University of Groningen, 9713 GZ Groningen, The Netherlands; Epistudia, 3008 Bern, Switzerland; Division of Nephrology, Department of Internal Medicine, University Medical Center Groningen, University of Groningen, 9713 GZ Groningen, The Netherlands

**Keywords:** glucose hemostasis, iron biomarkers, sex, type 2 diabetes

## Abstract

**Context:**

Sex-specific prevalence and incidence of type 2 diabetes (T2D) have been reported, but the underlying mechanisms are uncertain.

**Objective:**

In this study, we aimed to investigate whether iron biomarkers mediate the association between biological sex and glucose metabolism and the incidence of T2D.

**Methods:**

We used data from the general population enrolled in the prospective Prevention of REnal and Vascular ENd-stage Disease study in Groningen, The Netherlands. We measured ferritin, transferrin saturation (TSAT), hepcidin, soluble transferrin receptor (sTfR), fasting plasma glucose (FPG), fasting plasma insulin (FPI) levels, and incidence of T2D. We used multivariable regression and mediation analyses to investigate our hypothesis. All iron biomarkers, FPG, and FPI were log-transformed.

**Results:**

The mean (SD) age of the 5312 (51.3% female) individuals was 52.2 (11.6) years. Compared with males, females had lower FPG (β = −.01; 95% CI −0.02, −0.01) and FPI (β = −.03; 95% CI −0.05, −0.02) levels. Ferritin, hepcidin, and sTfR showed potential mediating effects on the association between sex and FPG, 21%, 5%, and 7.1%, respectively. Furthermore, these variables mediated 48.6%, 5.7%, and 3.1% of the association between sex and FPI, respectively. Alternatively, TSAT had a suppressive mediating role in the association of sex with FPG and FPI. The incidence of T2D was lower in females than in males (hazard ratio 0.58; 95% CI 0.44, 0.77), with 19.2% of this difference being mediated by ferritin.

**Conclusion:**

Iron biomarkers may partially mediate the association between sex and glucose homeostasis. Future studies addressing the causality of our findings are needed.

Type 2 diabetes (T2D) is a common metabolic disorder, and emerging evidence suggests that the incidence and prevalence of it differ by sex [1]. The prevalence of T2D is lower in females than males for the same age and the underlying mechanisms behind these biological sex differences are not well understood [2, 3]. Additionally, the prevalence of diabetes varies depending on the stage of reproductive life, with a lower prevalence before menopause in females [4].

Several studies suggest that iron metabolism indicators (eg, ferritin, hepcidin, soluble transferrin receptor [sTfR]) have roles in the development of diabetes [5-8]. Iron is a powerful pro-oxidant that raises the risk of diabetes by increasing reactive oxygen species (ROS) and oxidative stress, contributing to tissue damage [9]. The elevated level of serum ferritin, a biomarker of iron stores, is associated with glucose intolerance and insulin resistance, and is a risk factor for diabetes in healthy people [5, 10]. A Mendelian randomization (MR) study further suggested a causal role of iron metabolism in the development of T2D [11]. In line with these findings, clinical studies have shown that both iron chelation therapy and oral iron can alter glucose metabolism [12, 13].

Concerning iron parameters, the development of diabetes, and sex differences, studies up until now are rather inconsistent. Some studies indicate that ferritin levels are more strongly associated with diabetes in females compared with males [7, 14, 15]. These discrepancies in literature can potentially be explained by differences in iron according to sex, age, and menopause status. Levels of iron biomarkers are generally lower in females of fertile age. After menopause, the level of iron in the body may not reach the levels of iron stores that are found in men, but it increases substantially [16-19]. In addition, little is known about the mediating role of iron in the associations between sex and cardiometabolic risk factors [20], which underlines the need for more studies in this area. We therefore hypothesized that differences in iron parameters may partly explain the sex differences observed in T2D. We conducted the present study with the following objectives: (1) to evaluate cross-sectionally the association of sex with markers of glucose homeostasis; (2) to explore the longitudinal associations of sex with the incidence of T2D, and (3) to investigate the potential mediating role of iron biomarkers in the association of sex with glucose homeostasis and T2D.

## Methods and Materials

### Study Design

We used data from community-dwelling individuals enrolled in the prospective Prevention of REnal and Vascular ENd-stage Disease (PREVEND) study in Groningen, The Netherlands. Details have been described elsewhere [21]. Briefly, all inhabitants of Groningen aged 28 to 75 years (n = 85 421) were invited to participate between 1998 and 1999. The response rate was 47.8%. Pregnant women and patients with insulin-dependent diabetes were excluded. Individuals with a urinary albumin concentration ≥10 mg/L (n = 6000) and a randomly selected control group with urinary albumin concentration <10 mg/L (n = 2592), in total 8592 subjects, completed an extensive first screening. We used the second screening round data (2001-2003, n = 6894), since glucose, insulin, and iron biomarkers were available only for this survey. We excluded patients with missing data on glucose and insulin (n = 388), iron biomarkers (n = 560), prevalent T2D, a fasting glucose level of ≥7.0 mmol/L (126 mg/dL), a nonfasting glucose level of ≥11.1 mmol/L (200 mg/dL), or self-reported use of antidiabetic drugs (n = 437); iron supplement users (n = 27); and those with high-sensitivity C-reactive protein (hs-CRP) > 10 mg/L (n = 170). Hence, data from 5312 participants were used for the cross-sectional analysis. In prospective analysis, an additional 712 participants with missing data on the incidence of diabetes and those who were lost to follow-up were excluded (Fig. S1 [22]). The local institutional review board approved the PREVEND study protocol (MEC 96/01/022). All subjects provided written informed consent and study procedures were conducted according to the Declaration of Helsinki.

### Laboratory Measurements

A fasting blood sample was taken from each participant in the morning. All hematologic measurements were measured in fresh venous blood and stored immediately at −80 ˚C for further analysis. Serum ferritin was measured by immunoassay, serum iron by colorimetric assay, and serum transferrin by immunoturbidimetric assay (all Roche Diagnostics, Mannheim, Germany). Transferrin saturation (TSAT) was calculated as (serum iron ÷ (25× transferrin)) × 100 [23]. Serum hepcidin was measured with a competitive enzyme-linked immunosorbent assay with interassay and intra-assay coefficients of variation (CVs) of 16.2% and 8.6%, respectively [23]. An automated homogenous immunoturbidimetric assay with intra-assay and interassay CVs <2% and <5% respectively was used to quantify sTfR [23]. Fasting plasma glucose (FPG) was measured by dry chemistry (Eastman Kodak, Rochester, NY, USA) and fasting plasma insulin (FPI) with an immunoturbidometric assay (Diazyme Laboratories, Poway, CA, USA). We calculated the homeostasis model assessment of insulin resistance (HOMA-IR) as FPI (mU/L) × FPG (mmol/L)/22.5 and homeostasis model assessment of β-cell function (HOMA-B) as 20 × FPI (mU/L)/[FPG (mmol/L) − 3.5] [1]. HOMA-B and HOMA-IR values are estimates of β-cell function and insulin resistance of the glucose metabolism, respectively [1, 24]. Concentrations of total cholesterol were measured with standard methods. Creatinine-based estimated glomerular filtration rate (eGFR) was evaluated by Chronic Kidney Disease Epidemiology Collaboration equation [25]. Hemoglobin was measured using a Coulter Counter STKS sum (Coulter Corporation, Miami, FL, USA). hs-CRP was measured using nephelometry with a threshold of 0.175 mg/L and intra-assay and interassay CVs of <4.4% and 5.7%, respectively [26].

### Clinical Measurements

The height and weight of the participants were measured while standing, without shoes or heavy clothes. We considered sex a biological variable (rather than gender) and determined it as self-reported. Body mass index (BMI) was defined by dividing weight in kilograms by squared height in meters. Smoking (none, former, or current), alcohol consumption (abstinent, 1-4 units/month, 2-7 units/week, 1-3 units/day, or ≥3 units/day), and use of lipid-lowering and antihypertensive medications was based on self-reports [26]. Systolic blood pressure (SBP) was recorded based on the mean of 2 measurements. A history of myocardial infarction or stroke was investigated as the presence of cardiovascular disease (CVD) if the subject was hospitalized for at least 3 days due to that condition [21].

### Type 2 Diabetes Ascertainment

Follow-up time was defined as the period between the date of iron biomarkers measurement and the date of ascertainment of T2D, the date of the loss to follow-up, or the end of the follow-up period, which came first. T2D was determined if 1 or more of the following criteria were encountered: (1) FPG ≥7.0 mmol/L (126 mg/dL); (2) random sample plasma glucose ≥11.1 mmol/L (200 mg/dL); (3) self-reporting of physician diagnosis of diabetes; and (4) starting glucose-lowering medication use, obtained from a central pharmacy registration [1].

### Statistical Analyses

Baseline characteristics were expressed as mean (SD), median (25th-75th percentile), or count (%) for normally distributed, skewed, and categorical data, respectively. Characteristics of participants, according to sex differences, were investigated using an independent-sample t-test, Mann–Whitney U test, or chi-squared test, as appropriate. Skewed variables (iron biomarkers, FPG, FPI, HOMA-IR, and HOMA-β) were naturally log-transformed. Multiple linear regression models were used to assess the cross-sectional association of sex with iron biomarkers and the effect of sex and iron biomarkers on glucose, insulin, HOMA-IR, and HOMA-B. Multivariable Cox proportional hazards models were used to examine the effect of sex and iron biomarkers on incident T2D. Potential violation of the proportional hazard assumption was evaluated by using the Schoenfeld residuals test. No violations of the proportionality assumption were identified. All main analyses were adjusted for age (model 1) and additionally for BMI, alcohol, and smoking (model 2). We considered total cholesterol, use of lipid-lowering and antihypertensive drugs, hs-CRP, eGFR, SBP, CVD (model 3), and hemoglobin (model 4) as potential confounding and mediating factors in the association between sex, iron biomarkers, and glucose homeostasis, and included them in further exploratory analyses by adding these variables to model 2 (Fig. S2) [22]). Thus, model 2 was the main model in subsequent mediation analysis.

As a sensitivity analysis, we computed the absolute mean difference of iron biomarkers and glucose markers between males and females in model 2. Furthermore, we included physical activity measures in another sensitivity analysis within model 2, classified as ≤1 per week or >1 per week.

To account for and reduce potential bias due to missing data [27], multiple imputations of incomplete covariates using fully conditional specification were performed to obtain 5 complete data sets. Analyses were performed in each of the data sets and results were pooled using Rubin's rules [28]. Potential multicollinearity was ruled out by the assessment of variance inflation factors. Potential interactions between iron biomarkers and age were explored by adding the product term age and iron biomarkers into the main model. Natural cubic splines with 4 degrees of freedom were used to model the potential nonlinear effects of iron biomarkers on the incidence of diabetes. In sensitivity analyses, we investigated whether iron status can play a mediating role as a categorical variable. Thus, instead of using continuous iron biomarkers, were created a variable of body iron status based on clinical cut-offs; absolute iron deficiency (AID) as ferritin level ≤15 µg/L in females and ≤30 µg/L in males; iron overload as the fourth quartile of TSAT (30.7%) and ferritin (168 µg/L), and the rest of the participants as normal iron status [23, 29, 30].

#### Mediation analyses

Finally, we carried out mediation analyses based on ordinary least squares in linear models to evaluate if the sex differences in glucose homeostasis were mediated by the levels of iron biomarkers. In this method, X (sex) indicates the exposure variable, Y (glucose hemostasis) indicates the outcome variable, and M (iron biomarkers) indicates the mediator (Fig. S2 [22]). To conduct the mediation analyses, we specified the following paths: direct effect of the exposure on the outcome; indirect effect of the exposure, via a mediator on the outcome; total effect as the sum of direct effect and indirect effect; and proportion mediated (indirect effect/total effect) for biomarkers whose indirect and total effects were in the same direction [31]. To perform mediation analysis, the association of exposure with the mediator and the association of the mediator with the outcome should be statistically significant. Additionally, we used mediation analysis within a survival context to assess the mediating role of iron biomarkers when T2D is the outcome variable. In this study, we used the suppressive mediator role, when the indirect and total effects of the exposure variable on the outcome variable display opposite signs. A 2-sided *P* < .05 was considered to be statistically significant in all analyses. Data were analyzed using IBM SPSS software, version 27.0, PROCESS v4.1 by Andrew F. Hayes, and R software version 4.2.1 (R packages: survival and splines).

## Results

Baseline characteristics of the participants are shown in Table 1. Mean age was 52.2 (11.6) years, and 51.3% were female. Among 5312 participants at risk, 235 (4.4%) developed T2D during a follow-up of 7.3 (IQR 6.2-7.7) years. Median FPG and FPI levels were lower in females than in males. Females had significantly lower levels of HOMA-IR and higher levels of HOMA-B. Females were less likely to have T2D, CVD, and left ventricular hypertrophy. Compared with males, females had lower ferritin, hepcidin, sTfR, and TSAT concentrations (*P* < .001), and AID was more prevalent in females.

**Table 1.** Baseline characteristics of participants by sex

Characteristics	Total	Female	Male	*P* value
	n = 5312 (100%)	n = 2726 (51.3%)	n = 2586 (48.7%)	
Demographic and clinical				
Age (years)	52.2 ± 11.6	52 ± 11.3	53.5 ± 12.3	<.001
BMI, kg/m^2^	25.9 (23.5-28.7)	25.4 (22.9-28.5)	26.3 (24.2-28.7)	<.001
Systolic blood pressure, mmHg	122 (112-135)	116 (107.5-130)	127 (116.3-139)	<.001
Prevalent CVD (n, %)	282 (5.3)	85 (3.1)	197 (7.6)	<.001
Left ventricular hypertrophy (n, %)	183 (3.5)	69 (2.4)	114 (4.4)	<.001
Type 2 diabetes incidence (n, %)	235 (4.4)	90 (3.3)	145 (5.6)	<.001
Alcoholic behavior				
Abstinent	1254 (23.8)	817 (30.2)	437 (17.1)	<.001
1-4 units/month (n, %)	912 (17.3)	542 (20)	370 (14.5)	
2-7 units/week (n, %)	1703 (32.3)	818 (30.2)	885 (34.6)	
1-3 units/day (n, %)	1182 (22.4)	478 (17.6)	704 (27.5)	
≥4 units/day (n, %)	217 (4.1)	54 (2)	163 (6.4)	
Smoking behavior				
Never	1563 (29.7)	894 (33.1)	669 (26.2)	<.001
Former	2218 (42.2)	1040 (38.5)	1178 (46.1)	
Current	1473 (28)	766 (28.4)	707 (27.7)	
Use of antihypertensive drugs (n, %)	996 (18.8)	473 (17.4)	523 (20.3)	.007
Use of lipid-lowering drugs (n, %)	440 (8.3)	188 (6.9)	252 (9.8)	<.001
Laboratory				
Fasting plasma glucose, mmol/L	4.7 (4.4-5.2)	4.6 (4.3-5.1)	4.8 (4.5-5.3)	<.001
Fasting plasma Insulin, mU/L	7.9 (5.7-11.6)	7.5 (5.5-10.9)	8.4 (6-12.3)	<.001
Total cholesterol, mmol/L	5.4 (4.7-6.1)	5.4 (4.7-6.1)	5.4 (4.7-6.1)	.61
HOMA-IR, (mU/L^2^)/22.5	1.7 (1.2-2.6)	1.6 (1.1-2.4)	1.8 (1.3-2.8)	<.001
HOMA-B, %	132.3 (90.6-199.5)	135 (92.7-202.3)	131 (88.3-195.1)	.005
hs-CRP, mg/L	1.2 (0.6-2.6)	1.2 (0.6-2.8)	1.2 (0.6-2.5)	.036
eGFR, mL/min per 1.73 m^2^	92.1 (78.1-107.7)	106.0 (93.6-115.2)	79.9 (69.3-90.1)	<.001
TSAT (%)	24.3 (19.1-30.5)	23.1 (17.6-28.6)	26 (20.8-32.3)	<.001
Serum ferritin, µg/L	93 (46-167)	57 (29-106)	141 (85-225)	<.001
Serum hepcidin, nmol/L	3 (1.6-4.8)	2.3 (1.1-3.9)	3.7 (2.3-5.5)	<.001
Plasma sTfR, mg/L	2.5 (2.1-3)	2.4 (2-3)	2.5 (2.1-3)	.023
Hemoglobin, g/L	137.2 (128.9-146.6)	129.9 (124.1-135.3)	144.7 (138.6-151.5)	<.001
Iron status				
Normal group	4032 (75.9)	1946 (71.4)	2086 (80.7)	<.001
Absolute iron deficiency	799 (15)	693 (25.4)	106 (4.1)	
Iron overload	481 (9.1)	87 (3.2)	394 (15.2)	

Abbreviations: BMI, body mass index; CVD, cardiovascular disease; HOMA-IR, Homeostatic Model Assessment for Insulin Resistance; HOMA-B, homeostasis model assessment of β-cell function; eGFR, estimated glomerular filtration rate; hs-CRP, high-sensitivity C-reactive protein; TSAT transferrin saturation; sTfR, soluble transferrin receptor.

### Association Between Sex, Iron Biomarkers, and Glucose Homeostasis

In model 2, adjusted for age, BMI, smoking, and alcohol, (Table 2), females compared with males had lower levels of ferritin (β = −.36; 95% CI −0.38, −0.34), hepcidin (β = −.21; 95% CI −0.23, −0.19), sTfR (β = −.009; 95% CI −0.01, −0.005), and TSAT (β = −.07; 95% CI −0.08, −0.06). In models 1 and 2, females had lower levels of FPG, FPI, and HOMA-IR, and higher levels of HOMA-B than males (Table 3). Higher ferritin, hepcidin, and sTfR but lower TSAT were associated with higher FPG and FPI levels. Higher ferritin (β = .05; 95% CI 0.04, 0.07) and sTfR (β = .13; 95% CI 0.10, 0.18) and lower levels of TSAT (β = −.12, 95% CI −0.15, −0.08) were associated with higher HOMA-IR. Similarly, higher levels of ferritin and sTfR were associated with higher levels of HOMA-B (Table 3). Like the results above, females had lower levels of all markers except HOMA-B than males when looking at the absolute mean difference (Table S1 [22]).

**Table 2.** Comparison of iron biomarkers between sexes

	β (95% CI)*^c^*
Model 1*^a^*	
Ferritin, µg/L	−0.39 (−0.50, −0.37)
Hepcidin, nmol/L	−0.22 (−0.24, −0.20)
sTfR, mg/L	−0.003 (−0.007, 0.000)
TSAT, %	−0.07 (−.08, −0.06)
Model 2*^b^*	
Ferritin, µg/L	−0.36 (−0.38, −0.34)
Hepcidin, nmol/L	−0.21 (−0.23, −0.19)
sTfR, mg/L	−0.009 (−0.01, −0.005)
TSAT, %	−0.07 (−0.08, −0.06)

All iron biomarkers were log-transformed.

Abbreviations: sTfR, soluble transferrin receptor; TSAT, transferrin saturation.

^*a*^Model 1 was adjusted for age.

^*b*^Model 2 was adjusted for age, BMI, smoking, and alcohol use.

^*c*^Reference category for sex is males.

**Table 3.** Association between sex and iron biomarkers with markers of glucose homeostasis (insulin, FPG, FPI, HOMA-IR, and HOMA-B)

	Sex (ref = m) β (95% CI)	Ferritin (µg/L) β (95% CI)	Hepcidin (nmol/L) β (95% CI)	sTfR (mg/L) β (95% CI)	TSAT (%) β (95% CI)
Model 1*^a^*					
FPG	−0.02 (−0.02, −0.01)	0.01 (0.01, 0.02)	0.009 (0.005, 0.01)	0.02 (0.009, 0.03)	−0.03 (−0.04, −0.02)
FPI	−0.04 (−0.05, −0.03)	0.10 (0.08, 0.12)	0.06 (0.05, 0.08)	0.22 (0.18, 0.26)	−0.15 (−0.19, −0.12)
HOMA-IR	−0.06 (−0.07, −.04)	0.11 (0.09, 0.13)	0.07 (0.05, 0.09)	0.24 (0.20, 0.28)	−0.18 (−0.22, −0.14)
HOMA-B	0.02 (0.008, 0.04)	0.05 (0.03, 0.07)	0.03 (0.01, 0.05)	0.16 (0.11, 0.21)	−0.04 (−0.08, 0.003)
Model 2*^b^*					
FPG	−0.01 (−0.02, −0.01)	0.007 (0.004, 0.01)	0.003 (0.002, 0.005)	0.01 (0.003, 0.02)	−0.02 (−0.03, −0.02)
FPI	−0.03 (−0.05, −0.02)	0.05 (0.03, 0.06)	0.01 (0.006, 0.02)	0.13 (0.09, 0.16)	−0.09 (−0.12, −0.06)
HOMA-IR	−0.05 (−0.06, −0.04)	0.05 (0.04, 0.07)	0.01 (−0.001, 0.31)	0.13 (0.10, 0.18)	−0.12 (−0.15, −0.08)
HOMA-B	0.02 (0.005, 0.03)	0.02 (0.003, 0.04)	0.002 (−.02, 0.02)	0.09 (0.04, 0.13)	0.00 (−0.04, 0.04)

All iron biomarkers, FPG, FPI HOMA-IR, and HOMA-B were log-transformed.

Abbreviations: FPG, fasting plasma glucose; FPI, fasting plasma insulin; HOMA-B, homeostasis model assessment of β-cell function; HOMA-IR, Homeostatic Model Assessment for Insulin Resistance; sTfR, soluble transferrin receptor; TSAT, transferrin saturation.

^*a*^Model 1 was adjusted for age in the sex analysis and for both age and sex in the iron biomarkers analyses.

^*b*^Model 2 was adjusted for age, body mass index, smoking, and alcohol use in the sex analysis, and additionally for sex in the analyses on iron biomarkers.

### The Mediating Role of Iron Biomarkers in the Association Between Sex and Glucose Homeostasis

In the main model, the association of the female sex with lower glucose levels was mediated by ferritin, hepcidin, and sTfR (proportion mediated of 21%, 5%, and 7.1%, respectively), while TSAT suppressed this effect (Table 4). Similarly, ferritin, hepcidin, and sTfR mediated the association of sex with FPI by 48.6%, 5.7%, and 3.1%, respectively; however, TSAT suppressed this association. The mediating role of ferritin and sTfR on HOMA-IR was 40.3% and 2.4%, respectively, but TSAT had a suppressive mediating role. Hepcidin did not mediate the association of sex with HOMA-IR. Ferritin and sTfR had a suppressive mediating role on HOMA-B.

**Table 4.** Mediation analyses of iron biomarkers on the association between sex and FPG, FPI, insulin, HOMA-IR, and HOMA-B

	Model 1*^a^*			Model 2*^b^*		
	Indirect effect	Total effect	Proportion mediated	Indirect effect	Total effect	Proportion mediated
	β (95% CI)×10^−2^	β (95% CI)×10^−2^	(%)	β (95% CI)×10^−2^	β (95% CI)×10^−2^	(%)
Sex and FPG						
Ferritin, µg/L	−0.53 (−0.60, −0.47)	−2 (−18, −1.50)	26.5	−0.30 (−0.20, −0.30)	−1.40 (−1.50, −1.30)	21.0
Hepcidin, nmol/L	−0.20 (−0.24, −0.16)	−2 (−18, −1.50)	10.0	−0.07 (−0.11, −0.03)	−1.40 (−1.50, −1.30)	5.0
sTfR, mg/L	—	—	—	−0.10 (−0.2, −0.1)	−1.40 (−1.50, −1.30)	7.1
TSAT, %*^c^*	0.21 (0.19, 0.24)	−2 (−18, −1.50)	—	0.17 (0.15, 0.20)	−1.40 (−1.50, −1.30)	—
Sex and FPI						
Ferritin, µg/L	−3.75 (−4.48, −3.08)	−4.13 (−5.39, −2.86)	91	−1.70 (−2.00, −1.5)	−3.50 (−4.00, −3.10)	48.6
Hepcidin, nmol/L	−1.38 (−1.79, −0.99)	−4.13 (−5.39, −2.86)	33.4	−0.20 (−0.40, −0.10)	−3.50 (−4.00, −3.10)	5.7
sTfR, mg/L	—	—	—	−.11 (−.16, −.07)	−3.50 (−4.00, −3.10)	3.1
TSAT, %*^c^*	1.06 (0.79, 1.37)	−4.13 (−5.39, −2.86)	—	0.60 (0.50, 0.70)	−3.50 (−4.00, −3.10)	—
Sex and HOMA-IR						
Ferritin, µg/L	−4.35 (−5.11, −3.59)x	−5.84 (−7.22, −4.47)	74.5	−2.03 (−2.64, −1.42)	−5.03 (−6.23, −3.83)	40.3
Hepcidin, nmol/L	−1.62 (−2.07, −1.17)	−5.84 (−7.22, −4.47)	27.7	—	—	—
sTfR, mg/L	—	—	—	−0.12 (−0.27, −0.02)	−5.03 (−6.23, −3.83)	2.4
TSAT, %*^c^*	1.27 (0.96, 1.61)	−5.84 (−7.22, −4.47)	—	0.80 (0.55, 1.07)	−5.03 (−6.23, −3.83)	—
Sex and HOMA-B						
Ferritin, µg/L*^c^*	−1.91 (−2.68, −1.13)	2.33 (0.82, 3.85)	—	−0.79 (−1.5, −0.09)	1.95 (0.45, 3.45)	—
Hepcidin, nmol/L*^c^*	−0.74 (−1.2, −0.29)	2.33 (0.82, 3.85)	—	—	—	—
sTfR, mg/L*^c^*	—	—	—	−0.08 (−0.18, −0.01)	1.95 (0.45, 3.45)	—

All iron biomarkers, FPG, FPI HOMA-IR, and HOMA-B were log-transformed.

Abbreviations: FPG, fasting plasma glucose; FPI, fasting plasma insulin; HOMA-B, homeostasis model assessment of β-cell function; HOMA-IR, Homeostatic Model Assessment for Insulin Resistance; sTfRm soluble transferrin receptor; TSAT, transferrin saturation; .

^*a*^Model 1 was adjusted for age.

^*b*^Model 2 was adjusted for age, BMI, smoking, and alcohol use.

^*c*^Proportion mediated was not reported due to the different directions of indirect effect and total effect.

### Sex, Incidence of Type 2 Diabetes and the Mediating Role of Iron Biomarkers

In the main model, female sex was associated with a lower risk of T2D (HR 0.58; 95% CI 0.44, 0.77) (Table S2 [22]). TSAT was negatively associated with diabetes (HR 0.46; 95% CI 0.22, 0.94), but hepcidin and sTfR did not show any associations. When investigating nonlinearity, we observed a U-shaped association of ferritin with T2D (likelihood ratio test = 248.3, *P* < .001; Fig. S3 [22]). However, the association of ferritin with diabetes was significantly modified by age (*P*_int_ < .05). No significant interactions with age were observed for the other biomarkers.

In mediation analysis, the differences in ferritin levels between males and females were estimated to contribute to 19.2% of the additional cases of diabetes observed in males (Table S3) [22]). TSAT was not found to be a mediator.

### Association of Iron Status With Glucose Homeostasis

In model 2, using body iron status, AID, and iron overload vs the normal group, we found no association between iron status and glucose homeostasis or incidence of T2D (Table S4 [22]). Therefore, mediation analysis was not performed due to the lack of associations between the assumed mediators and the outcome.

### Additional Analyses

Analyses were repeated for all potential intermediate factors (ie, total cholesterol, hs-CRP, SBP, eGFR, lipid-lowering drugs, antihypertensive drugs, and CVD) in model 3 and model 4 (plus hemoglobin) (Tables S5-S8 [22]). In summary, in model 3, the effect sizes on the association between sex and iron biomarkers did not materially change, except for sTfR, while in model 4 sex was not associated with sTfR and TSAT (Table S5 [22]). In model 3, the association of sex with FPI disappeared, but after adjusting for hemoglobin (model 4), females had higher insulin levels (Table S6 [22]). Compared with the main model, the association between ferritin and glucose homeostasis did not change, but hepcidin became nonsignificant. STfR and TSAT preserved their associations in these models (except for nonsignificance of sTfR with FPG in model 4). The mediating role of ferritin between sex and glucose was maintained in both models. The mediating role of TSAT was also maintained in the association between sex with glucose and HOMA-IR in model 3. In model 4, unlike model 2, females show higher insulin, and, further, the mediating role of ferritin between sex and insulin becomes suppressive (Table S7 [22]). In models 3 and 4 the effect size between sex and iron biomarkers with incidence of T2D did not materially change (Table S8 [22]). Additional adjustment for physical activity as a potential confounder (categorized as once a week or less or more than once a week) in model 2 did not materially change the results (data not shown).

## Discussion

Our study investigated the potential mediating role of iron biomarkers on the association of sex with glucose homeostasis and the incidence of T2D. Females had lower levels of glucose and insulin than males, and these sex differences were partially explained by ferritin, hepcidin, sTfR, and TSAT. Ferritin could also play a mediating role in the association between female sex and the incidence of T2D. In addition, lower levels of ferritin and sTfR in females resulted in a greater reduction of HOMA-IR and HOMA-B compared with males.

### Association of sex and Glucose Hemostasis

In line with our study, previous evidence showed that females have lower glucose levels and better insulin sensitivity than males [2-4, 32]. Nuutila et al showed that whole-body insulin sensitivity was 41% greater in females than in males [33]. We used HOMA-IR and HOMA-B as intricately interdependent estimates of insulin resistance and β-cell function, respectively. Studies have shown the importance of pancreatic β-cell in the regulation of glucose metabolism and the prevention of diabetes [34, 35]. Our results agree with a previous study, which found HOMA-IR to be lower in females than in males [24]. Sheu et al found that adjusted HOMA-IR differed by sex and was positively correlated with ferritin in females. Finally, they concluded ferritin concentrations were related to the degree of insulin resistance [36].

### Association of Iron Biomarkers and Glucose Hemostasis

We found that females have lower levels of iron biomarkers than males. The lower levels of ferritin in females could be partly explained by their continual losses of iron in menstrual blood, pregnancies, and deliveries, but iron stores increase significantly after menopause. Some studies have shown an association between physical activity and iron biomarkers which differed between sexes. Zamelska et al in 2023 found that lower serum ferritin concentration concerns men with regular physical activity [37]. In our study, further adjustment for physical activity did not materially change our findings.

The role of iron has been proposed in the pathogenesis of chronic disorders like diabetes [18]. As observed in our study, epidemiologic observations have also demonstrated an association between elevated serum ferritin and the development of T2D in the general population [10, 14, 38]. A systematic meta-analysis of 15 prospective studies explored the association of higher ferritin with greater T2D risk, which appeared stronger among females than males [38]. Genetic evidence, using an MR study, supports a causal link between increased serum iron, ferritin, and TSAT with increased risk of T2D [11]. Inversely, Liang et al in a recent MR study showed that although a positive association of serum iron with T2D is possible, it is unlikely that iron biomarkers affect T2D [39]. The MR study would be especially interesting if the genes explain a substantial amount of variation. Otherwise, a very large sample size would be necessary to confidently establish a causal association. Although a genetic connection may not exist, our study demonstrated a clinical relationship. Additionally, it is important to recognize that MR studies, reliant on linear associations, may not yield the desired outcomes when non-linear relationships are at play, as we observed in our study [40].

One could assume that elevated serum ferritin levels may reflect systemic inflammation besides high iron stores [6]. However, ample evidence supports that high ferritin causes diabetes, and not vice versa [5, 41]. We excluded participants with hs-CRP >10 mg/L to account for inflammatory effects, and additionally adjusted for hs-CRP in models 3 and 4, which did not materially change the results.

Ferritin can induce insulin resistance and affect the risk of diabetes through several mechanisms. First, iron is a catalyst in the formation of ROS, which may be toxic for pancreatic β-cells and subsequently affect the synthesis and secretion of insulin [6, 14, 15]. We did not have available data on ROS to assess its role in insulin resistance. Furthermore, studies have shown a complex bidirectional relationship between iron metabolism with body fat, glucose, and lipid metabolism [42]. Excess iron may affect glucose uptake in adipose tissue by reducing glucose utilization in muscle, leading to insulin resistance [38]. Likewise, glucose, lipids, and insulin can affect iron regulatory pathways [42]. In addition, ferritin may interact with serum adiponectin, an insulin-sensitizing adipokine, in modulation of the risk of diabetes [43]. A prospective Finish Diabetes Prevention Study investigated an inverse association of adiponectin to ferritin ratio with diabetes risk, concluding an inverse association between serum ferritin and adiponectin [44]. We did not have body fat mass data in our study, but a detailed understanding of the mechanisms linking iron metabolism and metabolic risks should be suggested as a focus of future research.

Second, hemoglobin can also be involved, since in our study we found that after adjusting for hemoglobin, females showed higher insulin levels than males. A Chinese study showed that both higher hemoglobin and ferritin were associated with a high risk of diabetes, predominantly in females [45]. Elevated hemoglobin can increase blood viscosity, which can potentially decrease the supply of oxygen, glucose, and insulin to vital tissues, ultimately leading to insulin resistance [46].

Third, the “estrogen–iron” axis could also have implications for diabetes pathophysiology. Estrogen levels affect iron metabolism and may play a supportive role by increasing iron requirements in premenopausal and decreasing iron requirements in postmenopausal females [47]. High estrogen thus may also play a role in insulin sensitivity by balancing iron levels, since high iron can increase insulin resistance [2]. Our study did not have data on estrogen levels to explore whether estrogen could play a role in the association between sex, iron, and glucose homeostasis.

### Sex Differences in the Association Between Ferritin and Diabetes

Contradictory results have been published regarding sex differences in the association between ferritin and diabetes [14, 15, 36]. Some have only shown an association between ferritin and diabetes incidence in females, some in males, and others in both sexes [14, 15, 36, 48]. Kim and colleagues found in 13 848 adults that the age-adjusted odds ratio for diabetes in the fourth quartile of ferritin vs first was increased in both sexes [15]. In a study of 1444 ethnic Dutch, African, and South Asian Surinamese, and 162 Chinese patients with newly diagnosed T2D, ferritin was positively associated with T2D among females, but not in males [7, 14]. The discrepancies in results between studies could be due to differences in ferritin levels over time, which depend on sex, age, and menopausal status.

### Mediation Analysis

We investigated the mediating role of hepcidin, sTfR, and TSAT besides ferritin in the association of female sex with glucose homeostasis. Andrews et al found that the upper quartiles of hepcidin mRNA expression in obese men had an adjusted odd ratio for diabetes of 4.54 (CI 95% 0.95-21.66, *P* < .05) [9]. Hepcidin is a liver-derived 25 amino acid peptide and master regulator of iron homeostasis, by inhibiting the transport of iron into the blood circulation and also the recycling of iron from macrophages [6]. Elevated hepcidin has been shown to potentially lead to iron accumulation in the liver and increased oxidative stress [5]. In a meta-analysis, the sTfR:ferritin ratio was inversely correlated with the risk of diabetes. In only 1 study, an association between high sTfR, as a sensitive indicator of high metabolic demand for iron, and an increased risk of diabetes was identified [5]. There is much debate about TSAT, with some studies reporting a high TSAT, while others have shown both high and low TSAT to be associated with diabetes [49]. Our study showed however that TSAT may have a suppressive role in the association between female sex and glucose hemostasis. Iron deficiency can also impair insulin expression and lead to metabolic changes through association with obesity. In this context, the inflammatory state associated with excessive adipose tissue may decrease iron absorption by increasing proinflammatory cytokines upregulating hepcidin [49]. In our study, we did not identify an association between AID or high iron status, and outcomes, most likely due to the low power of the categorized groups.

### Clinical Implications in Diabetes Management

A better understanding of sex differences in physiology and disease and the identification of underlying mechanisms may lead to better prevention and treatment. Our observational study indicates the role of iron in understanding sex differences in diabetes. Future studies should explore our hypothesis more and investigate causality in diverse populations. In addition, from a clinical and public health perspective, further exploring our results could provide new insights into glycemic management and guiding sex-specific therapeutic interventions for the treatment of diabetes.

### Strengths and Limitations

To our knowledge, this is the first study investigating the potential mediating role of iron biomarkers on the association of sex with glucose hemostasis. Our study presents several strengths, including a long follow-up time, a comprehensive set of iron biomarkers, and an analysis of multiple potential confounders. Our study also has some limitations. The cross-sectional nature of the study hampers drawing any conclusions on causality. Since iron parameters were measured at 1 point during this cohort, we could not evaluate the possibility of changes in iron parameters longitudinally. It should be realized that most epidemiologic studies use a single baseline measurement for studying the association of variables with outcomes, which adversely affects the strength and significance of the association of these variables with outcomes. If intra-individual variability of variables is considered, this results in the strengthening of associations that also existed for single measurements of these variables [50]. Since the study sample was mainly composed of white individuals, the findings might not generalize to other populations and should be replicated with different groups. We were not able to assess dietary iron intake patterns and sex hormones, and supplementary studies are needed to take these parameters into account. We did not have information on menopausal status, which precluded our possibility to explore the role of menopause on the investigated associations; however, no interaction with age, except for ferritin, was identified in our study. In addition, reverse causality could be present in the analysis between iron biomarkers and diabetes, but the exclusion of the first 3 months of follow-up did not change our results.

## Data Availability

The datasets analyzed in the current research are available upon reasonable request from the corresponding author and are not publicly available.

## Abbreviations

AID: absolute iron deficiency
BMI: body mass index
CV: coefficient of variation
CVD: cardiovascular disease
eGFR: estimated glomerular filtration rate
FPG: fasting plasma glucose
FPI: fasting plasma insulin
HOMA-IR: homeostasis model assessment of insulin resistance
hs-CRP: high-sensitivity C-reactive protein
MR: Mendelian randomization
ROS: reactive oxygen species
SBP: systolic blood pressure
sTfR: soluble transferrin receptor
T2D: type 2 diabetes
TSAT: transferrin saturation
