# Correction to: “Potential Mediating Role of Iron Biomarkers in the Association of Sex With Glucose, Insulin, and Type 2 Diabetes”

**DOI:** 10.1210/jendso/bvae133

**Published:** 2024-07-18

In the above-named article by Khatami F, Lange T, Groothof D, Ahanchi NS, Quezada-Pinedo HG, Raeisi-Dehkordi H, De Borst MH, Vidal P-M, Mohan S, Prabhakaran D, Bano A, Bakker SJL, Muka T, and Eisenga MF (J Endo Society. 2024; 8(7); doi: 10.1210/jendso/bvae098), there was an error in Table 3 and in the author list.

In the originally published article, in Table 3, footnote A read as, “Model 1 was adjusted for age.” In the corrected article, footnote A reads as, “Model 1 was adjusted for age in the sex analysis and for both age and sex in the iron biomarkers analyses.” In Table 3, footnote B read as, “Model 2 was adjusted for age, body mass index, smoking, and alcohol use.” In the corrected article, footnote B reads as, “Model 2 was adjusted for age, body mass index, smoking, and alcohol use in the sex analysis, and additionally for sex in the analyses on iron biomarkers.” In Table 3, footnote C, “Analyses of iron biomarkers were additionally adjusted for sex” was accidently added to the table. In the corrected Table 3, footnote C, “Analyses of iron biomarkers were additionally adjusted for sex” is omitted.

In the author list, the author Sailesh Mohan was incorrectly listed as Mohan Sailesh.

The article has been corrected online.

doi: 10.1210/jendso/bvae098

